# A Chinese soil conservation dataset preventing soil water erosion from 1992 to 2019

**DOI:** 10.1038/s41597-023-02246-4

**Published:** 2023-05-26

**Authors:** Jialei Li, Hongbin He, Qinghua Zeng, Liding Chen, Ranhao Sun

**Affiliations:** 1 Research Center for Eco-Environmental Sciences, Chinese Academy of Sciences, Beijing, 100085 China; 2 University of Chinese Academy of Sciences, Beijing, 100190 China

**Keywords:** Ecosystem services, Geomorphology

## Abstract

Soil conservation service (SC) is defined as the ability of terrestrial ecosystems to control soil erosion and protect soil function. A long-term and high-resolution estimation of SC is urgent for ecological assessment and land management on a large scale. Here, a 300-m resolution Chinese soil conservation dataset (CSCD) from 1992 to 2019, for the first time, is established based on the Revised Universal Soil Loss Equation (RUSLE) model. The RUSLE modelling was conducted based on five key parameters, including the rainfall erosivity (interpolation of daily rainfall), land cover management (provincial data), conservation practices (weighted by terrain and crop types), topography (30 m), and soil properties (250 m). The dataset agrees with previous measurements in all basins (R^2^ > 0.5) and other regional simulations. Compared with current studies, the dataset has long-term, large-scale, and relatively high-resolution characteristics. This dataset will serve as a base to open out the mechanism of SC variations in China and could help assess the ecological effects of land management policies.

## Background & Summary

Excessive soil erosion can negatively impact crop production, carbon transfer, soil organisms, and soil nutrition^1–4^. To prevent soil erosion, many countries have attempted to improve soil conservation services, including China. China governments have made a lot of plans and policies to control soil erosion for recent years, for example, legislation for soil and water conservation, building agricultural terraces^5,6^, 3-North Shelter Forest Program (since 1978)^7^, and Conversion of Farmland to Forests and Grass (Grain-for-Green) (since 1999)^8^. Recent research shows that China has led in a quarter of the global increase in greening after 2000^9^. However, China supports one-fifth of the world population with 9% of the total global cropland^10^, which means that the reduction of cropland has a potential to affect China grain security^11,12^.

Over the past few decades, China has experienced an expansion of farmland, a massive growing total population (from 987 million people in 1980 to 1.413 billion people in 2020), and a large urbanization process^13–15^. Although these increasing trends have slowed down in recent years, anthropogenic activities, along with climate change, have brought about non-negligible changes in soil erosion risk and soil conservation service. To support the huge population, agriculture may face the challenges to be expanded or intensified^16,17^. Either approach puts pressure on the land, including soil fertility and water conservation^18^. To weight the relationship between human and the land, a reliable dataset of soil conservation service in China is urgent.

High-precision, long-term soil conservation service data are requested in China. However, accurate and high-precision soil conservation service datasets based on field measurement are unfeasible and money-consuming on a national scale. The Chinese government has decided to conduct the third national soil census from 2022 to 2025^19^; however, this would be an arduous task and hard to reflect long-term changes. Modelled soil conservation service data could meet these demands and be more feasible, which were usually based on modelling soil water erosion prevented by vegetation and practice measures^20^.

Development of soil erosion models based on remote-sensing data and GIS technology makes it possible to assess soil conservation captivity and its dynamic changes. By far the most widely used empirical model to simulate soil erosion is the Revised Universal Soil Loss Equation (RUSLE) model, which predicts longtime average annual soil loss in specified experimental fields^21,22^. They describe the relationship between soil loss rate and the following effects: soil properties, topography, vegetation cover, land management, and rainfall and runoff^23^. In order to apply the USLE-family model on a large scale, many researchers have made improvements and enhancements to the parameters of the model^23–29^. Yang *et al*.^30^ develop a GIS-based RUSLE model to offer a pioneering overview of global soil erosion on cell grids. Now the USLE-based models have been used to estimate soil erosion on regional and global scales^31–34^. They were also embedded in the InVEST model to compute the Sediment Retention Index^35^. Although there were some uncertainties in the accuracy of global soil erosion estimation based on USLE-family models^36–38^, considering the availability of data, model mechanism, and China’s geographical conditions, we finally chose the RUSLE model. Currently, soil conservation and soil erosion data in China are mostly based on regional or watershed scales^39–42^, only few studies are based on national scales^43,44^. These national-scale studies were only for one year or average year data, lacking long-term series data.

Based on high-precision and long-term series data, this study calibrated the input factors and obtained the database of soil conservation service and soil erosion rate in China from 1992 to 2019. These results can provide data support for assessing soil erosion risk in China and drive analysis of past changes to enhance regional management. The main goal of this data is to analyze the interannual changes in soil conservation in China and provide a basis for identifying potential soil erosion hotspots. In addition, this dataset can be regarded as a comparison of soil conservation for other studies on different scales in the future. The results can provide a reference for further improvement of model parameters and lay a foundation for identifying driving factors of soil erosion changes.

## Methods

We used the RUSLE (Revised Universal Soil Loss Equation) model to estimate the soil erosion rate in China during 1992–2019, and certain adjustments were made in the factor calculation according to the actual backgrounds in China. The model equations are as follows:

1 $${\rm{S}}{{\rm{E}}}_{{\rm{P}}}=R\cdot L\cdot S\cdot K$$

2 $${\rm{S}}{{\rm{E}}}_{{\rm{a}}}=R\cdot L\cdot S\cdot K\cdot C\cdot P$$

where SE_*p*_ is the predicted potential annual soil erosion on bare land (t ha^−1^ a^−1^), SE_a_ is the predicted actual annual soil erosion (t ha^–1^ a^–1^) on land with vegetation cover and erosion control practices, *R* is the rainfall erosivity factor (MJ mm ha^–1^ h^–1^ a^–1^), *LS* is the topographic factor (dimensionless) with *L* being the slope length factor and *S* being the slope factor, *K* is the soil erodibility factor (t h MJ^–1^ mm^–1^), *C* is the vegetation cover and management factor (dimensionless), and *P* is the support practice factor (dimensionless). Every factor was calculated according to the original resolution of the input data (for example, DEM data for 30 m × 30 m, soil properties data for 250 m × 250 m), then all the factors were resampled to a resolution of 300 m × 300 m by bilinear interpolation and multiplied to obtain the soil erosion rate in each year.

In this study, soil conservation service (SC, t ha^–1^ a^–1^) is defined as soil retention, which is soil water erosion prevented by vegetation and practice measures, equal to potential soil erosion minus actual soil erosion:

3 $${\rm{S}}{\rm{C}}={\rm{S}}{{\rm{E}}}_{{\rm{p}}}-{\rm{S}}{{\rm{E}}}_{{\rm{a}}}=R\cdot L\cdot S\cdot K\cdot \left(1-C\cdot P\right)$$

**Fig. 1:**
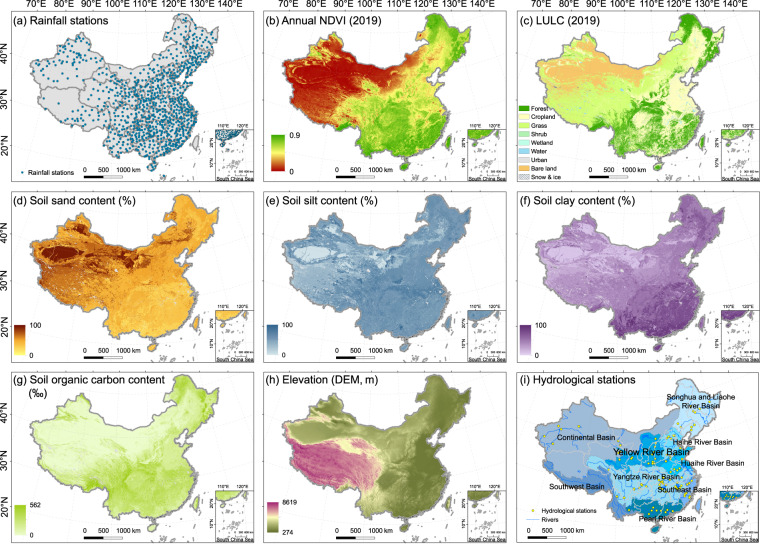
Raw data to develop the soil conservation dataset. NDVI: Normalized difference vegetation index. LULC: Land use and land cover types.

**Fig. 2:**
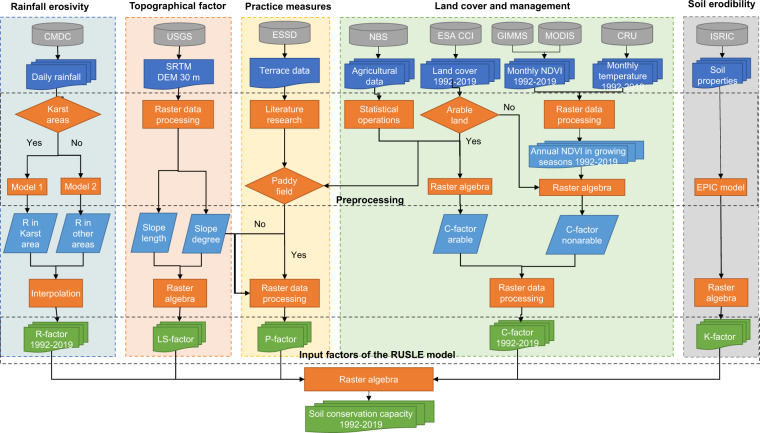
Flow chart of the methods and data sources.

The list of the input data is shown in Table 1, and the spatial distribution of the raw data are shown in Fig. 1. The framework to develop the dataset is shown in Fig. 2.

**Table 1:** Input data.

Data name	Spatial resolution	Format	Temporal period	Source
NASA SRTM digital elevation	30 m	grid	Static (2000)	Shuttle Radar Topography Mission digital elevation model (SRTM DEM) data
Rainfall data	Station	txt	Daily 1992–2019	China Meteorological Data Service Centre (CMDC) (http://data.cma.cn/en)
Normalized difference vegetation index (NDVI)	8 km	tiff	Half-monthly 1992–2015	Global Agricultural Monitoring System (GIMMS) AVHRR NDVI3g datasets
	0.05°	tiff	Half-monthly 2016–2019	Terra MODIS NDVI data (MOD13C2 V6)
Land temperature	0.5°	tiff	Monthly 1992–2019	Climatic Research Unit (CRU TS)
Land cover	300 m	tiff	Annual 1992–2019	European Space Agency Climate Change Initiative Land Cover (ESA CCI LC)
Soli properties	250 m	tiff	Static	International Soil Reference and Information Centre (ISRIC) (http://data.isric.org/geonetwork/srv/chi/catalog.search;jsessionid=A887E5B4)
Sown areas of major farm crops	Provincial	csv	Annual 1992–2019	National Bureau of Statistics of China (NBS) Database (https://data.stats.gov.cn)

### Estimation of the *R*-factor

The calculation of the *R*-factor is essential in the RUSLE model because it can reflect the impact of natural rainfall on soil erosion, which is especially sensitive to climate change. According to the USLE manual, the R-factor calculation requires the storm kinetic energy (E) and maximum 30-min rainfall intensity (I_30_). A recent study uses hourly rainfall data to estimate the *R*-factor^45^, which contains average rainfall erosivity data from 1951 to 2018 in China. However, it is difficult to obtain continuous, high-precision, and full-coverage rainfall records in long-term estimations of the R-factor on a national scale. Considering the feasibility, large-scale studies generally use monthly or annual rainfall data to calculate the *R*-factor; however, this method can bring many uncertainties and inaccuracies^46^. Here, to better describe China’s long-term annual rainfall erosivity, we made a trade-off and used a wildly accepted daily rainfall erosivity model developed according to climate characteristics in China^47^:

4 $$R={\sum }_{j=1}^{24}{R}_{i}$$

5 $${R}_{i}=\alpha {\sum }_{j=1}^{k}{\left({P}_{j}\right)}^{\beta }$$

6 $$\beta =0.8363+\frac{18.77}{{P}_{d12}}+\frac{24.455}{{P}_{y12}}$$

7 $$\alpha =21.586{\beta }^{-7.1892}$$

where *R* is the annual rainfall erosivity, and *R*_*i*_ is the half monthly rainfall erosivity. *P*_*j*_ is the daily erosive rainfall amount on the *j*-th day during the half-month (only select the days with *P*_*j*_ ≥ 12 mm, which is the threshold of a rainfall erosivity event in China^48^). *P*_*d12*_ represents the average daily rainfall (mm) with a daily rainfall of 12 mm or more, and *P*_*y12*_ represents the average annual rainfall with a daily rainfall of 12 mm or more.

Although the above model is the most widely used method in China, there are some improved models that is more applicable in some specific regions^48,49^. Especially it overestimated rainfall erosion in the karst areas of southwest China due to the special geological background there^49^. Therefore, we searched for a more suitable model for the karst region and finally selected a power law equation with a sinusoidal relationship reflecting seasonal variations of rainfall based on daily data^50^. It has proven to be that the performance of this model is good in karst areas of China^49^. The equation is as follows:

8 $${R}_{d}={\rm{0}}{\rm{.2686}}\,\left[{\rm{1}}+0.5412\,{\rm{\cos }}\left(\frac{\pi }{6}j-\frac{{\rm{7\pi }}}{6}\right)\right]{P}_{d}^{{\rm{1}}{\rm{.7263}}}$$

where *R*_*d*_ is the daily rainfall erosivity in the month *j* (MJ·mm·ha^−1^·h^−1^), *P*_*d*_ is the daily rainfall (mm) in the day *d*, and the sum of the *R*_*d*_ in a year is the annual rainfall erosivity.

The source of the daily rainfall data and the distribution of meteorological stations are shown in Table 1 and Fig. 1a, respectively. Annual values of the *R*-factor were firstly calculated for each station, then the values were interpolated to erosivity maps employing the method of Universal Kriging, which has proven to be an effective method for the spatial interpolation of the rainfall erosivity in China^44,51^.

### Modification of the *C*-factor

The *C*-factor is closely related to the types of vegetation and crops^25^; therefore, the values of the *C*-factor in arable and non-arable land in China were calculated separately. Some adjustments were made to the classification of crop types and the reference *C*-values of these crop types in arable land^25,52^ according to the actual agricultural conditions in China (Table 2). The released crops were classified into ten categories according to the main crop types in each province issued by the National Bureau of Statistics. The values of the *C*-factor in arable land (*C*_*A*_) were calculated by the following equation:

9 $${C}_{A}={\sum }_{{\rm{n}}={\rm{1}}}^{{\rm{10}}}{C}_{{cropn}}\times {{Region}}_{{Cropn}}$$

where *C*_*cropn*_ represents the value of the *C*-factor of the crop type *n*, and *Region*_*Cropn*_ represents the share of the sown areas of this crop type *n* in the total agricultural land in a province.

**Table 2:** The values of the *C*-factor in cropland (*C*_*cropn*_).

Farm crops			n	*C*_*crop*_
Grain crops	Cereal	Rice	1	0.15
		Corn	2	0.38
		Other cereals	3	0.2
	Beans		4	0.32
Tubers			5	0.34
Sugar crops	Sugarcane and Beetroots		6	0.15
Oil-bearing crops			7	0.25
Fiber crops	Cotton		8	0.4
	Other fiber crops		9	0.28
Tobacco			10	0.5
Vegetables			11	0.25
Medicinal materials			12	0.15
Succulence			13	0.1
Orchards	Grapes		14	0.35
	Melons and fruits orchards		15	0.25
	Other orchards		16	0.32
Other farm crops			17	0.15

**Fig. 3:**
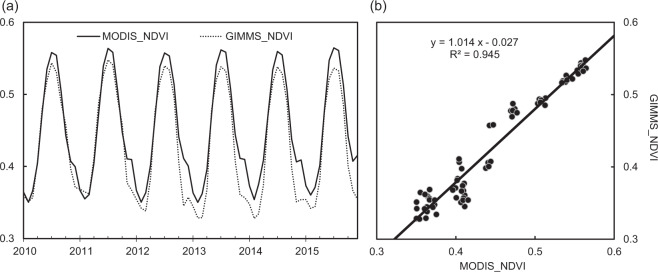
Comparison of the NDVI from GIMMS with MODIS based on temporal fluctuations (**a**) and correlative relationship (**b**).

The values of the *C*-factor in non-arable land (*C*_*NA*_) were estimated by vegetation coverage (based on the NDVI data) and reference values of the *C*-factor of various land cover types. The GIMMS NDVI products were the main source of the NDVI data from 1992 to 2015 for data integrity; however, they were unavailable after 2015. To form a temporally continuous SC dataset covering 1992–2019, we searched for other monthly NDVI data covering 2016–2019. We compared the MODIS NDVI with GIMMS NDVI with the temporal span overlapping from January 2010 to December 2015. We found that the temporal fluctuations of the monthly NDVI were consistent, and their correlation was obvious (R^2^ = 0.95, *p* < 0.01) (Fig. 3). Therefore, the MODIS NDVI was selected to supplement the NDVI data after 2015. The monthly NDVI was then normalized using the following equation^52^:

10 $${F}_{cover}=\frac{NDV{I}_{i}-{\rm{\min }}(NDV{I}_{n})}{{\rm{\max }}(NDV{I}_{n})-{\rm{\min }}(NDV{I}_{n})}$$

where *F*_*cover*_ is the vegetation coverage, which is the monthly average value of the NDVI during the growing season (the months with a mean surface air temperature ≥ 0 °C) (Fig. 1b)^53^. *NDVI*_*i*_ means the value of the NDVI in grid cell *i*, and max(*NDVI*_*n*_) and min(*NDVI*_*n*_) represent the maximum and minimum values of the NDVI in year *n*, respectively. The values of the *C*_*NA*_ were calculated by^25,52^:

11 $${C}_{NA}={\rm{\min }}\left({C}_{NAn}\right)+\left[{\rm{\max }}\left({C}_{NAn}\right)-{\rm{\min }}\left({C}_{NAn}\right)\right]\times \left(1-{F}_{cover}\right)$$

where C_*NAn*_ is the reference values of the *C*-factor in land cover type *n* (Table 3) (Fig. 1c), and max(*C*_*NAn*_) and min(C_*NAn*_) are the maximum and minimum value of the C_*NAn*_, respectively.

**Table 3:** The values of the C-factor in non-cropland (*C*_*NAn*_).

n	Land cover type	*C*_*NAn*_ (maximum-minimum)
1	Broadleaf evergreen forest	0.0001–0.003
2	Broadleaf deciduous forest	0.0001–0.003
3	Needleleaf evergreen forest	0.0001–0.003
4	Needleleaf deciduous forest	0.0001–0.003
5	Mixed forest	0.0001–0.003
6	Tree open	0.01–0.15
7	Shrub	0.01–0.15
8	Herbaceous	0.01–0.15
9	Herbaceous with sparse tree/shrub	0.01–0.15
10	Sparse vegetation	0.1–0.5
11	Wetland	No data
12	Bare land	0.1–0.5
13	Urban	No data
14	Snow and ice	No data

### Improvement of the *P*-factor

The P-factor reflects the efficacy of support practices that humans made to control soil erosion with a range of 0 (no soil water erosion) to 1 (no support practices)^21,22^. However, the *P*-factor is difficult to quantify in large-scale modelling of soil erosion^54^. At present, the large-scale quantification of the *P*-factor is mainly based on literature analysis^54–56^, which can only show statistical data without time changing. In this study, we have mapped and refined the distribution of the *P*-factor by an unprecedented method. We separated paddy and terraced fields from other land use types. Then we assigned different *P*-factor values according to different terrains based on a meta-analysis of *P* values of support practices^54^. The horizontal paddy field was assigned a value of 0.2. *P*-factor values in other arable areas were assigned according to the slope (Table 4), which was only applied on the terraces because terraces are one of the most common and identifiable support practices in mountainous regions in China^5,54^. For other non-arable lands, the value of the *P*-factor is 1 because support practices are mainly built on croplands in China^56^.

**Table 4:** The values of the *P*-factor for cropland on terraces.

Slope gradient	The values of the *P*-factor
<4.6°	0.45
4.6° ~ 7°	0.52
7° ~ 9°	0.61
9° ~ 14°	0.70
14° ~ 24°	0.92
>24°	1

### Calculation of the *LS*- and *K*-factor

The *LS*-factor reflects the natural geographic influence of topography on soil erosion. The *LS*-factor consists of a slope length (*L*) factor and a slope steepness (*S*) factor, which are calculated by DEM data in GIS-based RUSLE model^21,22^. The resolution of the DEM data influences the accuracy of the simulation of the *LS*-factor, as it has significant scaling effect^57–59^. Considering the trade-off between the accuracy of simulations and feasibility of calculation, we used the DEM data with a resolution of 30 m × 30 m (Fig. 1h). The formulas for calculating the *L*-factor are as follows^21,60^:

12 $$L={\left(\frac{{l}_{i}}{{\rm{22}}{\rm{.13}}}\right)}^{m},$$

13 $${l}_{i}={\sum }_{1}^{i}\frac{{D}_{i}}{{\rm{\cos }}{\theta }_{i}}-{\sum }_{1}^{{\rm{i}}-1}\frac{{D}_{i}}{{\rm{\cos }}{\theta }_{i}}=\frac{{D}_{i}}{{\rm{\cos }}{\theta }_{i}},$$

14 $$m=\left\{\begin{array}{c}\begin{array}{c}0.2,\beta  < 1 \% \\ {\rm{0}}{\rm{.3}},1 \% \le \beta  < 3 \% \\ {\rm{0}}{\rm{.4}},3 \% \le \beta  < 5 \% \\ {\rm{0}}{\rm{.5}},\beta \ge 5 \% \end{array}\end{array}\right.,$$

where *m* is the slope length exponent, *l*_*i*_ is the slope length of a grid *i*, *D*_*i*_ is the horizontal projection distance of the slope length of each grid along the runoff direction, *θ*_*i*_ is the angle of the slope of a grid *i*, and *β* is the slope gradients in %.

The *S*-factor was calculated by following the method in the CSLE model, which is improved according to the different slope degree in China^61^:

15 $$S=\left\{\begin{array}{c}{\rm{10}}{\rm{.80}}\;{\rm{\sin }}\theta +{\rm{0}}{\rm{.03,}}\theta  < {5}^{\circ }\\ {\rm{16}}{\rm{.80}}\;{\rm{\sin }}\theta -0.5{\rm{,}}{5}^{\circ }\le \theta  < 1{0}^{\circ }\\ {\rm{21}}{\rm{.91}}\;{\rm{\sin }}\theta -0.96{\rm{,}}\theta \ge 1{0}^{\circ }\end{array}\right.,$$

where *θ* is the slope angle in degrees.

Soil erodibility (*K*-factor) reflects the soil’s resistance to both detachment and transportation, which is originally measured by establishing unit plot for each soil type^21^. Sharpley and Williams^62^ establish regression equations between the measured data of plot experiments and soil properties in the EPIC model (Sharpley and Williams 1990), which make it feasible to calculate the *K*-factor on a large scale^30,63,64^. The formula is as follows:

$$K=\left\{{\rm{0}}{\rm{.2}}+{\rm{0}}{\rm{.3}}\exp \left[-{\rm{0}}{\rm{.0256}}* SAN\left({\rm{1}}-\frac{SIL}{{\rm{100}}}\right)\right]\right\}$$

16 $${\left(\frac{SIL}{CLA+SIL}\right)}^{{\rm{0}}{\rm{.3}}}\left({\rm{1}}-\frac{{\rm{0}}{\rm{.25}}* {OC}}{{\rm{OC}}+\exp \left(3.72-2.95* OC\right)}\right)$$

$$\left(1-\frac{0.7{\rm{SN}}1}{SN1+\exp \left(-5.51+22.9SN1\right)}\right)$$

17 $$SN1=1-\frac{SAN}{100}$$

where *SAN*, *SIL*, *CLA* and *OC* are the percentage content of sand, silt, clay and organic carbon from topsoil, respectively (Fig. 1d–g).

### Change detection

A linear regression was employed to quantify long-term changes of the *R*-factor, *C*-factor, and SC from 1992 to 2019. The least squares were applied to calculate annual change rates, and the equation is as follows:

18 $${\theta }_{slope}=\frac{n\times {\sum }_{i=1}^{n}{i}\times {A}_{i}-{\sum }_{i=1}^{n}i{\sum }_{i={\rm{1}}}^{n}{A}_{i}}{n\times {\sum }_{i=1}^{n}{i}^{2}-{\left({\sum }_{i=1}^{n}i\right)}^{2}},$$

where *ϑ*_*slope*_ is the regression coefficient (slope of the linear regression), representing the annual changing rate of the data. *A*_*i*_ represents the data in the year of *i*.

## Data Records

The dataset of soil conservation service preventing soil water erosion in China (1992–2019) is available at Science Data Bank (https://cstr.cn/31253.11.sciencedb.07135). Data 10.57760/sciencedb.07135^65^. This dataset includes nine zip files (“.rar”). All the data in the zip files are raster data (“.tif”). The details about every zip file were as follows.

- “C1992–2019.rar”: It contains the mean value and changing rate of the C-factor from 1992 to 2019, which were named “C_mean.tif” and “C_slope.tif”, respectively.
- “C_year.rar”: The detailed data of the C-factor in two-year increments from 1992 to 2019. The raster data were named “Cyyyy_300.tif”; “yyyy” is the year and “300” is the resolution of the data (300 m × 300 m resolution).
- “K_300.rar”: It contains the K-factor data, named “K_300.tif” (300 m × 300 m resolution).
- “LS_300.rar”: It contains the LS-factor data, named “LS_300.tif” (300 m × 300 m resolution).
- “P_300.rar”: It contains the P-factor data, named “P_300.tif” (300 m × 300 m resolution).
- “R1992–2019.rar”: The mean value and changing rate of the R-factor in China from 1992 to 2019, named “R_mean.tif” and “R_slope.tif”, respectively. The resolution of the data is 1 km × 1 km.
- “R_year.rar”: The R-factor data in two-year increments from 1992 to 2019 (1 km × 1 km resolution). The naming of the data is as “Ryyyy.tif”, and “yyyy” is the year.
- “SC1992–2019.rar”: The mean value and changing rate of soil conservation service (300 m × 300 m resolution).
- “SC_year.rar”: SC data in two-year increments from 1992 to 2019 (300 m × 300 m resolution).

## Technical Validation

Since soil conservation service is calculated based on soil erosion rates estimated by the RUSLE model, its reliability can be indirectly validated by verifying simulated soil erosion^39,42,43^. However, it is difficult to obtain abundant measured soil erosion rates from plot experiments for large-scale validation of modelled soil erosion, so we applied a cross-comparison of the assessments with substituted observations and other regional simulations of soil erosion^52^. Substituted observations refer to observed sediment runoff data for hydrologic stations in eight major river basins in China from 2010 to 2019 (Fig. 1i)^66^, which were compared with modelled soil erosion rates by computing the correlation coefficients (R^2^) between the mean values of them in every basin. High values of R^2^ usually mean satisfactory estimations of soil erosion^67–69^. We also compared assessments of soil erosion rates with other regional simulations using the USLE-based models, which can verify whether our results are in a reliable range. We also presented a correlation analysis between our estimated soil conservation service and other regional estimations that applying USLE-based models. The sources of collected studies are in the supplementary Tables S1, S2.

**Fig. 4:**
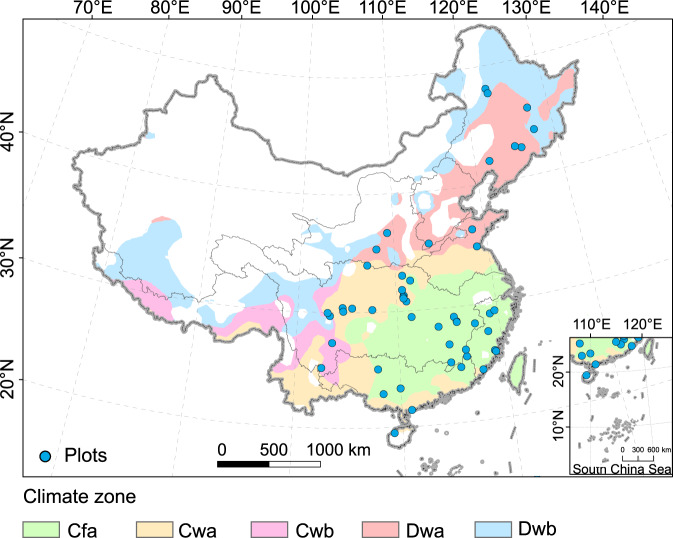
Distribution of the collected plot data and climate zones in China. C: temperate, D: cold, f: without dry season, w: dry winter, a: hot summer, b: warm summer.

Furthermore, because the development of the RUSLE model is based on multiple regression of soil loss from the unit plot data, it was necessary to compare our results with the plot measurements. We used measured data representing 957 plot years in China from published literature to verify our results^69^. The distribution of the collected plots is shown in Fig. 4. To compare the results, we grouped the measured data according to climate zones and land cover types. The land use types used included cropland, grassland, shrubland, and forest land, and the climate zones included Cfa, Cwa, Cwb, Dwa, and Dwb (the naming of the climate zones follows the Köppen-Geiger climate classification^70^). Then the grouped measures data were compared with the average annual simulated data in these grouped areas. Because the principle of acquiring measured and simulated data was different, they are unsuitable for direct comparison. Therefore, if data distributions of the two types of results were similar, it could be considered that the simulated data can reflect the distribution difference of the actual data.

**Fig. 5:**
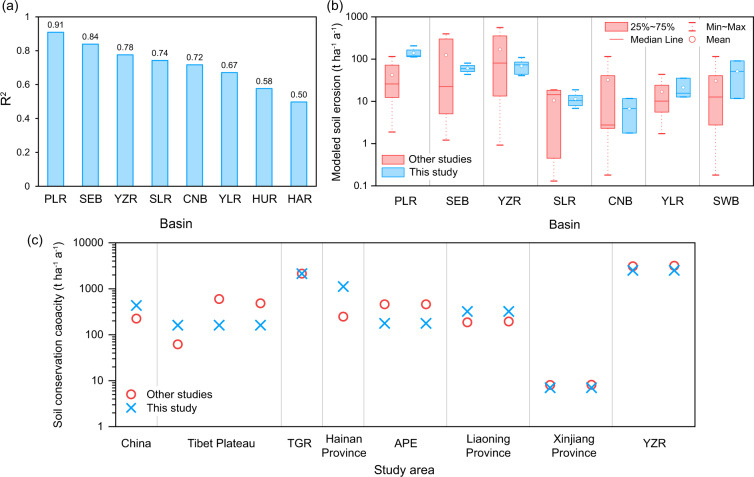
(**a**) Correlation (R^2^) between measurements of sediment runoff and simulations of soil erosion. (**b**) Comparison of simulated annual soil water erosion rates among different studies using USLE-based models. PLR: Pearl River Basin, SEB: Southeast Basin, YZR: Yangtze River Basin, SLR: Songhua and Liaohe River Basin, CNB: Continental Basin, YLR: Yellow River Basin, HAR: Haihe River Basin, HUR: Huaihe River Basin, SWB: Southwest Basin. (**c**) Comparison of soil conservation service from this study with those from other studies. TGR: Three Gorges Reservoir region, APE: Agro-pastoral ecotone of northern China.

The comparison between the plot data and the modelled data (Fig. 5a) shows that the simulation of soil erosion can be accepted (all R^2^ ≥ 0.5). The simulation in the Pearl Basin has the highest correlation with the sediment runoff data (R^2^ = 0.91), while the correlation is lowest in the Haihe River Basin (R^2^ = 0.50). In addition, modelled soil erosion results in this study were compared with those in other studies representing seven basins in China (Fig. 5b and Table S1), which indicates that our simulations of soil erosion are within a reliable range. However, our modelled soil erosion rates are overestimated compared with those from collected literature in the Pearl River Basin. We also collected some SC data from other studies on a regional scale (Table S2). Results of comparison with other regional SC (Fig. 5c) indicates that our SC data in these regions are consistent with these results. The model uncertainty is mainly derived from different algorithms of the parameters and data sources^71^, which is also the reason for the differences between our results and other results.

**Fig. 6:**
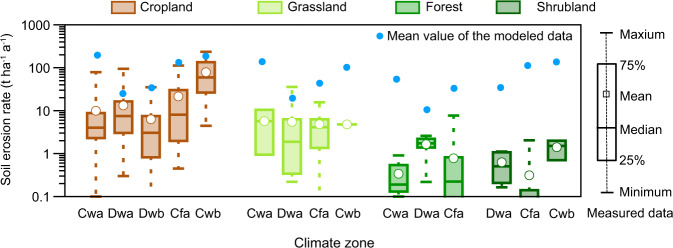
Comparison between soil erosion rates simulated by the model and those measured on runoff plots.

The comparison between measured and simulated results indicated that modeled soil erosion was generally higher than measured soil erosion (Fig. 6). The simulation results in cropland were closer to the measured values than those in natural vegetation (grassland, forest, and shrubland). However, the gap between the simulated and the measured results of each land cover type in the Cwa climate zone was larger than in other climate zones. The main reason may be that the Cwa climate zone is mainly in regions with extreme rainfall and changeable meteorological conditions, such as China’s southern and southwestern edges, where the measured data may be insufficient and cannot represent annual soil erosion well. Although there were some differences between the simulated and the measured results, the trend of data distribution was similar among different land cover types and climatic zones. The comparisons demonstrated that that the dataset could reflect the difference in soil erosion and conservation among different climatic zones and land cover types in China. The prediction of the variation trend in soil erosion and conservation was close to the actual situation. These areas usually lack sufficient measured data for model-fitting research^38,69^, so there was a need for more references for model correction.

Although these data have been improved by using relatively high-resolution input data and calibrating model factors, some limitations still remain. First, there are certain difficulties in the trade-off between the feasibility of calculation and the accuracy of the simulations in large-scale modelling. The use of the RUSLE model has a spatial scale effect on the precision of the input data^72^, which is one of the reasons that lead to the differences between large-scale model estimations (our study) and small-scale estimations (other studies). The finer input data can produce more reliable results. We will employ finer input data in further improvements; for example, simulations of the *R*-factor could be improved based on 30-min or finer rainfall data. Second, further exploration of factor calculation methods that are applicable to different regions is still needed. For example, the *R*-factor in high mountain areas (such as the Tibetan Plateau) can be improved using more suitable methods^48^. Lastly, this dataset still needs further validations of every factor; for example, the K-factor estimation based on the EPIC model could be compared with measured data in some specific areas. However, the lack of sufficient measurement data for comparison disturbs the subsequent dataset verification.

## Supplementary information

Supplementary

## Data Availability

The computing progress was completed in the ArcGIS 10.6 software. No custom code was used.
