# Supplementary material for: A Chinese soil conservation dataset preventing soil water erosion from 1992 to 2019
Source: Sci Data. 2023 May 26;10:319. doi: 10.1038/s41597-023-02246-4 (PMC10220090; doi:10.1038/s41597-023-02246-4)
Supplement: Supplementary file 1 — Supplementary [file 41597_2023_2246_MOESM1_ESM.docx]

**Supplementary**

**Table S1.** Overview of modelled soil erosion rates (SE) from collected studies compared with our results.

| Study area | Other studies |  |  | This study |  |  |
| --- | --- | --- | --- | --- | --- | --- |
|  | Time series | Average SE (t·ha^–1^·a^–1^) | Resolution* | Time series | Average SE (t·ha^–1^·a^–1^) | Resolution |
| Yangtze River Basin | | | | | | |
| Jiangxi Province | 2001, 2015 | 63.75–70.42 (Zhou et al., 2018) | 1 km | 1992-2019 | 40.69–77.44 (2001, 2015) | 300 m |
|  | 2010 | 353.54 (Chen et al., 2014) | 1 km |  | 73.31 |  |
|  | 2000, 2005, 2010, 2015 | 90.60 (Li et al., 2021) | 1 km |  | 73.31 |  |
|  | Average of 2002–2016 | 0.92 (Teng et al., 2019) | 1 km |  | 48.01 |  |
| Hunan Province | 2010 | 558.24 (Chen et al., 2014) | 1 km |  | 109.01 |  |
| The southern hill region of China | 2010 | 421.81 (Chen et al., 2014) | 1 km |  | 84.86 |  |
|  | 2000, 2005, 2010, 2015 | 108.20 (Li et al., 2021) | 1 km |  |  |  |
| South of Gansu and northwest of Sichuan | 2015 | 13.39 (Wei et al., 2021a) | 1 km |  | 43.65 |  |
|  | 2000, 2005, 2010, 2015 | 11.53 (Li et al., 2021) | 1 km |  |  |  |
| Southeast Basin | | |  |  | |  |
| Fujian Province | 2010 | 298.71 (Chen et al., 2014) | 1 km | 1992-2019 | 80.47 | 300 m |
|  | Average of 2002–2016 | 5.07 (Teng et al., 2019) | 1 km |  | 60.35 |  |
|  | 1990, 2000, 2010, 2015 | 15-30 (Zeng et al., 2017) | 30 m (DEM) |  |  |  |
| Zhejiang Province | 2010 | 395.02 (Chen et al., 2014) | 1 km |  | 58.01 |  |
|  | Average of 2002–2016 | 1.21 (Teng et al., 2019) | 1 km |  | 43.39 |  |
| Pearl River Basin | | |  |  | |  |
| Guangdong Province | 2012 | 22.94 (Gao et al., 2017) | 500 m | 1992-2019 | 116.42 | 300 m |
|  | Average of 2002–2016 | 1.88 (Teng et al., 2019) | 1 km |  | 112.63 |  |
|  | 2000, 2005, 2010, 2015 | 115.19 (Li et al., 2021) | 1 km |  | 122.88 |  |
| Guizhou Province | 2002 | 28.7 (Xu et al., 2008) | 25 m |  | 206.66 |  |
| Yellow River Basin | | |  |  | |  |
| Loess Plateau | 2008 | 24.05 (Fu et al., 2011) | 30 m (DEM) | 1992-2019 | 15.56 | 300 m |
|  | Average of 1991-1995 | 10.13 (Jin et al., 2021) | 30 m |  |  |  |
|  | Average of 2011-2015 | 5.95 (Jin et al., 2021) | 30 m |  |  |  |
|  | 2000, 2005, 2010, 2015 | 8.27 (Li et al., 2021) | 1 km |  |  |  |
| Gansu Province | - | 15.00 (Zhang and McBean, 2016) |  |  | 12.07 |  |
|  | Average of 2002–2016 | 1.72 (Teng et al., 2019) | 1 km |  |  |  |
| Shaanxi Province | 2006 | 41.74–43.34 (Cheng et al., 2009) |  |  | 35.31 |  |
|  | Average of 2002–2016 | 2.78 (Teng et al., 2019) | 1 km |  |  |  |
| Continental Basin | | |  |  | |  |
| Three-North Shelter Forest Region | 2015 | 2.30 (Ji et al., 2018) |  | 1992-2019 | 1.78 | 300 m |
| Tibet Plateau | Average of 1990–2000 | 40.5 (Xiao et al., 2003) | 1 km |  | 11.67 |  |
|  | Average of 2002–2016 | 2.76 (Teng et al., 2018) | 1 km |  |  |  |
|  | - | 0.18–114.98 (Wei et al., 2021b) | - |  |  |  |
| Songhua and Liaohe River Basin | | |  |  | |  |
| Liaoning Province | Average of 2016–2020 | 18.73 (Li, 2022) | 1 km | 1992-2019 | 18.84 | 300 m |
|  | Average of 2002–2016 | 0.45 (Teng et al., 2019) | 1 km |  |  |  |
| Jilin Province | 2005 | 17.70 (Zhao, 2018) | 90 m |  | 11.71 |  |
|  | 2010 | 18.25 (Zhao, 2018) | 90 m |  | 13.91 |  |
|  | 2015 | 14.33 (Zhao, 2018) | 90 m |  | 7.92 |  |
|  | Average of 2002–2016 | 0.13 (Teng et al., 2019) | 1 km |  | 9.13 |  |
| Black Soil Region | 1980, 1990, 2000, 2010, 2017 | 3.91–4.45 (mean=4.22) (Fang and Fan, 2020) | 90 m (DEM) |  | 6.82 |  |
| China | | |  |  | |  |
|  | 2001, 2012 | 6.42 (Borrelli et al., 2017) | 25 km | 1992-2019 | 25.78 | 300 m |
|  | 2000, 2005, 2010, 2015 | 35.98 (Li et al., 2021) | 1 km |  | 30.40 |  |
|  | Average of 2002–2016 | 1.44 (Teng et al., 2019) | 1 km |  |  |  |
|  | 2018 | 64.88–81.75 (Zhuang et al., 2021) | 10 km |  | 34.16 |  |

* The data resolution refers to the resolution of final results of soil erosion estimation. If this information is missed, it will be replaced by the resolution of the DEM data.

**Table S2.** Overview of collected soil conservation capacity (SC) in different regions and their sources compared with our results.

| Study area | Other studies | | | This study | | |
| --- | --- | --- | --- | --- | --- | --- |
|  | Time | Average SC (t ha^–1^ a^–1^) | Resolution* | Time | Average SC (t ha^–1^ a^–1^) | Resolution |
| China | 2009 | 224.42 (Rao et al., 2013) | 90 m | 1992-2019 | 433.45 | 300 m |
| Tibet Plateau | Average of 1990–2000 | 61.9–598.3 (mean value=483.03)  (Xiao et al., 2003) | 1 km |  | 161.35 |  |
| Three Gorges Reservoir region of China | 2000–2010 | 2134.73 (Xiao et al., 2017) | 90 m |  | 2132.81 |  |
| Hainan Island | 2008 | 247.28  (Rao et al., 2013) | 90 m (DEM) |  | 1117.01 |  |
| Agro-pastoral ecotone of northern China | 2000, 2008 | 459.03–459.40 (Liu et al., 2021) | 90 m (DEM) |  | 177.09 |  |
| Liaoning Province | Average of 2016–2020 | 186.15–194.35 (Li, 2022) | 1 km |  | 321.43 |  |
| Xinjiang Province | 1996–2012 | 8.09–8.17 (Ma et al., 2020) | 1 km |  | 7.05 |  |
| Yangtze River Basin | 2000, 2015 | 3082–3164 (Kong et al., 2018) | 90 m (DEM) |  | 2515.09 |  |

* The data resolution refers to the resolution of final results of soil conservation capacity. If this information is missed, it will be replaced by the resolution of the DEM data.

**Reference:**

Borrelli, P., Robinson, D. A., Fleischer, L. R., Lugato, E., Ballabio, C., Alewell, C., Meusburger, K., Modugno, S., Schutt, B., Ferro, V., Bagarello, V., Oost, K. V., Montanarella, L., and Panagos, P.: An assessment of the global impact of 21st century land use change on soil erosion, Nature Communications, 8, 1-13, <https://doi.org/10.1038/s41467-017-02142-7>, 2017.

Chen, S., Yang, X., Xiao, L., and Cai, H.: Study of soil erosion in the southern hillside area of China based on RUSLE model, Resources Science, 36, 1288-1297, 2014.

Cheng, L., Yang, Q., Xie, H., Wang, C., and Guo, W.: GIS and CSLE based quantitative assessment of soil erosion in Shaanxi, China, Journal of Soil and Water Conservation, 23, 61-66, <https://doi.org/10.13870/j.cnki.stbcxb.2009.05.022>, 2009.

Fang, H. Y., and Fan, Z. M.: Assessment of Soil Erosion at Multiple Spatial Scales Following Land Use Changes in 1980-2017 in the Black Soil Region, (NE) China, International Journal of Environmental Research and Public Health, 17, <https://doi.org/10.3390/ijerph17207378>, 2020.

Fu, B., Liu, Y., Lu, Y., He, C., Zeng, Y., and Wu, B.: Assessing the soil erosion control service of ecosystems change in the Loess Plateau of China, Ecological Complexity, 8, 284-293, <https://doi.org/10.1016/j.ecocom.2011.07.003>, 2011.

Gao, F., Wang, Y. P., and Yang, J. X.: Assessing soil erosion using USLE model and MODIS data in the Guangdong, China, 3rd International Symposium on Earth Observation for Arid and Semi-Arid Environments, 74, <https://doi.org/10.1088/1755-1315/74/1/012007>, 2017.

Ji, C., Li, X., Jia, Y., and Wang, L.: Dynamic Assessment of Soil Water Erosion in the Three-North Shelter Forest Region of China from 1980 to 2015, Eurasian Soil Science, 51, 1533-1546, <https://doi.org/10.1134/s1064229318120050>, 2018.

Jin, F. M., Yang, W. C., Fu, J. X., and Li, Z.: Effects of vegetation and climate on the changes of soil erosion in the Loess Plateau of China, Science of The Total Environment, 773, <https://doi.org/10.1016/j.scitotenv.2021.145514>, 2021.

Kong, L., Zheng, H., Rao, E., Xiao, Y., Ouyang, Z., and Li, C.: Evaluating indirect and direct effects of eco-restoration policy on soil conservation service in Yangtze River Basin, Science of The Total Environment, 631-632, 887-894, <https://doi.org/https://doi.org/10.1016/j.scitotenv.2018.03.117>, 2018.

Li, J. L., Sun, R. H., Xiong, M. Q., and Chen, L. D.: Methodology of Time Series of Soil Erosion Dataset in Water Erosion Area of China in Five-year Increments (2000-2015), Journal of Global Change Data & Discovery, 5, 203-212, 2021.

Li, M.: Evaluation of functional importance of soil and water conservation in Liaoning Province, Heilongjiang Hydraulic Science and Technology, 50, 215-218, <https://doi.org/10.14122/j.cnki.hskj.2022.02.059>, 2022.

Liu, M., Zhang, H., Ren, H., and Pei, H.: Spatiotemporal Variations of the Soil Conservation in the Agro-pastoral Ecotone of Northern China Under Grain for Green Program, Research of Soil and Water Conservation, 28, 172-178, <https://doi.org/10.13869/j.cnki.rswc.2021.05.020>, 2021.

Ma, X., Zhu, J., Yan, W., and Zhao, C.: Assessment of soil conservation services of four river basins in Central Asia under global warming scenarios, Geoderma, 375, 114533, <https://doi.org/10.1016/j.geoderma.2020.114533>, 2020.

Rao, E., Xiao, Y., Ouyang, Z., and Zheng, H.: Spatial characteristics of soil conservation service and its impact factors in Hainan Island, Acta Ecologica Sinica, 33, 746-755, 2013.

Teng, H., Liang, Z., Chen, S., Liu, Y., Viscarra Rossel, R. A., Chappell, A., Yu, W., and Shi, Z.: Current and future assessments of soil erosion by water on the Tibetan Plateau based on RUSLE and CMIP5 climate models, Science of The Total Environment, 635, 673-686, <https://doi.org/https://doi.org/10.1016/j.scitotenv.2018.04.146>, 2018.

Teng, H. F., Hu, J., Zhou, Y., Zhou, L. Q., and Shi, Z.: Modelling and mapping soil erosion potential in China, Journal of Integrative Agriculture, 18, 251-264, <https://doi.org/10.1016/s2095-3119(18)62045-3>, 2019.

Wei, J., Li, C., Wu, L., Xie, X., Lv, J., and Zhou, X.: Study on soil erosion in Northwestern Sichuan and Southern Gansu (NSSG) based on USLE, Journal of Soil and Water Conservation, 35, 31-37+46, 2021a.

Wei, M., Fu, S., and Liu, B.: Quantitative research of water erosion in the Qinghai-Tibet Plateau, Advances in Earth Science, 1-13, 2021b.

Xiao, Q., Hu, D., and Xiao, Y.: Assessing changes in soil conservation ecosystem services and causal factors in the Three Gorges Reservoir region of China, Journal of Cleaner Production, 163, S172-S180, <https://doi.org/https://doi.org/10.1016/j.jclepro.2016.09.012>, 2017.

Xiao, Y., Xie, G., and An, K.: The function and economic value of soil conservation of ecosystems in Qinghai-Tibet Plateau, Acta Ecologica Sinica, 2367-2378, 2003.

Xu, Y.-Q., Shao, X.-M., Kong, X.-B., Peng, J., and Cai, Y.-L.: Adapting the RUSLE and GIS to model soil erosion risk in a mountains karst watershed, Guizhou Province, China, Environmental Monitoring and Assessment, 141, 275-286, <https://doi.org/10.1007/s10661-007-9894-9>, 2008.

Zeng, S., Wang, Z., Wang, X., Lin, J., Chen, X., and Chen, S.: Temporal and spatial variation of soil erosion in fujian province, Bulletin of Soil and Water Conservation, 37, 305-312+320, <https://doi.org/10.13961/j.cnki.stbctb.2017.06.050>, 2017.

Zhang, C., and McBean, E. A.: Estimation of desertification risk from soil erosion: a case study for Gansu Province, China, Stochastic Environmental Research and Risk Assessment, 30, 2215-2229, <https://doi.org/10.1007/s00477-015-1186-2>, 2016.

Zhao, M. Quantitative study on hydraulic soil erosion in Jilin province based on RUSLE model. Master. China University of Geosciences (Beijing ).2018.

Zhou, X., Ma, G., Cao, G., Yu, F., Zhou, Y., Jia, Q., and Zhang, Y.: Soil erosion changes in Jiangxi Province from 2001 to 2015 based on USLE model, Bulletin of Soil and Water Conservation, 38, 8-11+17+12, 2018.

Zhuang, H., Wang, Y., Liu, H., Wang, S., Zhang, W., Zhang, S., and Dai, Q.: Large-Scale Soil Erosion Estimation Considering Vegetation Growth Cycle, Land, 10, 473, 2021.
